# Implementation outcomes of the integrated district evidence to action (IDEAs) program to reduce neonatal mortality in central Mozambique: an application of the RE-AIM evaluation framework

**DOI:** 10.1186/s12913-024-10638-4

**Published:** 2024-02-02

**Authors:** Aneth Dinis, Quinhas Fernandes, Bradley H Wagenaar, Sarah Gimbel, Bryan J Weiner, Grace John-Stewart, Ermyas Birru, Stephen Gloyd, Ruth Etzioni, Dorlim Uetela, Isaías Ramiro, Artur Gremu, Orvalho Augusto, Stélio Tembe, Jaime L Mário, Jalilo E Chinai, Alfredo F Covele, Cassimo M Sáide, Nélia Manaca, Kenneth Sherr

**Affiliations:** 1 National Department of Public Health, Ministry of Health, Maputo City, Mozambique; 2 Department of Global Health, University of Washington, Seattle, WA USA; 3 Department of Epidemiology, University of Washington, Seattle, WA USA; 4 Department of Child, Family & Population Health Nursing, University of Washington, Seattle, WA USA; 5 Department of Medicine, University of Washington, Seattle, WA USA; 6 Department of Pediatrics, University of Washington, Seattle, WA USA; 7 Department of Biostatistics, University of Washington, Seattle, WA USA; 8 Department of Health Systems and Population Health, University of Washington, Seattle, WA USA; 9 National Institute of Health, Maputo, Mozambique; 10 Comité para Saúde de Moçambique, Maputo, Mozambique; 11 Eduardo Mondlane University, Maputo, Mozambique; 12 Tete Provincial Health Service, Tete, Mozambique; 13 Zambezia Provincial Health Service, Quelimane, Mozambique; 14 Department of Industrial and Systems Engineering, University of Washington, Seattle, WA USA

**Keywords:** Audit & Feedback, Implementation outcomes, RE-AIM, Neonatal mortality, Mozambique, Maternal and Child Health, Implementation science, Health Systems Research

## Abstract

**Background:**

Scarce evidence exists on audit and feedback implementation processes in low-resource health systems. The Integrated District Evidence to Action (IDEAs) is a multi-component audit and feedback strategy designed to improve the implementation of maternal and child guidelines in Mozambique. We report IDEAs implementation outcomes.

**Methods:**

IDEAs was implemented in 154 health facilities across 12 districts in Manica and Sofala provinces between 2016 and 2020 and evaluated using a quasi-experimental design guided by the Reach, Effectiveness, Adoption, Implementation, and Maintenance (RE-AIM) framework. Reach is the proportion of pregnant women attending IDEAs facilities. Adoption is the proportion of facilities initiating audit and feedback meetings. Implementation is the fidelity to the strategy components, including readiness assessments, meetings (frequency, participation, action plan development), and targeted financial support and supervision. Maintenance is the sustainment at 12, 24, and 54 months.

**Results:**

Across both provinces, 56% of facilities were exposed to IDEAs (target 57%). Sixty-nine and 73% of pregnant women attended those facilities’ first and fourth antenatal consultations (target 70%). All facilities adopted the intervention. 99% of the expected meetings occurred with an average interval of 5.9 out of 6 months. Participation of maternal and child managers was high, with 3076 attending meetings, of which 64% were from the facility, 29% from the district, and 7% from the province level. 97% of expected action plans were created, and 41 specific problems were identified. “Weak diagnosis or management of obstetric complications” was identified as the main problem, and “actions to reinforce norms and protocols” was the dominant subcategory of micro-interventions selected. Fidelity to semiannual readiness assessments was low (52% of expected facilities), and in completing micro-interventions (17% were completed). Ninety-six and 95% of facilities sustained the intervention at 12 and 24 months, respectively, and 71% had completed nine cycles at 54 months.

**Conclusion:**

Maternal and child managers can lead audit and feedback processes in primary health care in Mozambique with high reach, adoption, and maintenance. The IDEAs strategy should be adapted to promote higher fidelity around implementing action plans and conducting readiness assessments. Adding effectiveness to these findings will help to inform strategy scale-up.

**Supplementary Information:**

The online version contains supplementary material available at 10.1186/s12913-024-10638-4.

## Background

The Sustainable Development Goals call to end preventable deaths of newborns and children under five, with all countries aiming to reduce neonatal mortality to 12 or fewer deaths per 1,000 live births and under-five mortality to 25 or fewer deaths per 1,000 births by 2030 [1]. In 2020, more than 5 million children died before age five, and 47% of those deaths occurred during the neonatal period—the first 28 days of life—even without the increase in mortality attributable to COVID-19 [1]. Sub-Saharan Africa has the highest neonatal mortality rate in the world at 27 deaths per 1,000 live births, contributing 43% of the global share of neonatal deaths [1]. In Mozambique, a country with limited resources and a high disease burden, neonatal mortality remains a significant public health problem, with a rate of 28 deaths per 1,000 live births in 2020 [1].

Research suggests that increasing the coverage and quality of preconception, antenatal, intrapartum, and postnatal evidence-based interventions globally by 2025 could avert 71% of neonatal deaths, saving approximately two million lives per year at a low-cost [2]. Furthermore, available interventions can reduce the major cause of neonatal mortality—preterm, intrapartum, and infections-related deaths —by 58%, 79%, and 84%, respectively [2]. Examples of these interventions include maternal immunization, screening and management of infections, preventive treatments for malaria, emergency obstetric care (EmOC), and immediate procedures for neonatal care [2, 3].

Countries with high neonatal mortality, including Mozambique, have clinical practice guidelines to implement these interventions along the continuum of care in health facilities and at the community level. Despite these guidelines, implementation is inconsistent, with only 47% of recommended care provided by providers [4, 5]. Additionally, the coverage and quality of evidence-based interventions are uneven in low-resource countries, primarily due to poor service readiness (lack of financial, material, and human resources), lack of provider training, weak provider awareness of current clinical guidelines [4, 6, 7], lack of accountability of provider performance, and poor leadership and management capacity [8, 9].

The utilization of maternal and child health services in Mozambique is high. In 2015, an estimated 93% of pregnant women attended a first antenatal care (ANC) visit, 73% gave birth in a health facility, and 76% of children ages 12–23 months received the third pentavalent vaccination [10]. Compliance with clinical guidelines can be improved by implementing structural changes and strengthening leadership and management across different health system levels [11, 12], facilitating improvements in health service utilization and neonatal outcomes.

Audit and feedback (A&F) is an evidence-based implementation strategy used in healthcare settings to systematically evaluate individual professional practice or performance based on targets or standards and improve health professionals’ compliance with guidelines [13]. However, more evidence is needed on how to use A&F most effectively [14]. Most studies on A&F use a randomized controlled trial design and have been conducted in high-income settings and demonstrate small to moderate effectiveness [13, 15, 16]. Uncertainty remains about the potential impact of A&F in low-income settings where the disease burden is higher, health systems are weaker, and baseline implementation of clinical practice guidelines has considerable space for improvement. Further evidence generation is needed from these settings, where more significant effects can be expected.

The Integrated District Evidence to Action (IDEAs) program is a multi-component A&F implementation strategy that aims to improve the coverage and quality of a bundle of existing evidence-based interventions targeting major causes of neonatal mortality. Funded by the Doris Duke Charitable Foundation and the National Institutes of Health, IDEAs was implemented between October 2016 and December 2020 in two provinces across 12 districts in central Mozambique. The goal was to determine the effectiveness of the IDEAs intervention strategy, led by district management teams, to serve as a foundation for national scale-up.

The Reach, Effectiveness, Adoption, Implementation, and Maintenance (RE-AIM) framework is one of the most frequently applied implementation science frameworks [16–18]. RE-AIM was developed to guide research in complex real-world settings and has great potential to provide detailed, nuanced information on whether and how quality improvement interventions succeed [19, 20]. Reach describes the absolute number, proportion, and representativeness of individuals willing to participate in the intervention. Effectiveness describes the impact on selected outcomes. Adoption captures the absolute number, proportion, and representativeness of settings and intervention agents willing to initiate a program. Implementation represents the intervention agents’ fidelity to the components of an intervention’s protocol, including consistency of delivery as intended, time spent, and associated costs. Maintenance refers to the extent to which a program or policy becomes institutionalized or part of routine organizational practices and policies [18, 19]. Guided by the RE-AIM framework, we report on the IDEAs A&F implementation process and outcomes in this article. We hope our findings will help inform and improve the IDEAs program’s future replication, adaptation, or scale-up.

## Methods

### Program description

The IDEAs program is designed to improve health service delivery by identifying clinical performance gaps and enabling maternal and child (MCH) nurses to monitor, evaluate, prioritize, and adapt solutions to improve compliance with Ministry of Health (MOH) guidelines targeting major causes of neonatal mortality. IDEAs is a multi-component implementation strategy that conducts routine health service readiness assessments (SRAs); applies an A&F process that engages MCH managers at facility, district, and province levels to review performance and develop solutions that address identified gaps; and supports ongoing district-to-facility supportive supervision and provision of flexible funding to support implementation of these identified solutions (***micro-interventions***, defined here as solutions selected, implemented, or adapted at the health facility level, not requiring significant financial resources). IDEAs cycles are iterative, repeated every six months.

### Steps involved in the IDEAs implementation strategy

#### Step 1: facility and district service readiness assessment

Before the semi-annual A&F meeting, standardized assessment tools ( the World Health Organization Service Availability and Readiness Assessment -SARA [21], program questionaries, observation, and registry review) were applied in three randomly selected, rotating facilities within each district to assess (1) structural readiness to deliver perinatal clinical intervention, including staffing levels, training, availability of essential commodities, equipment and supplies, and data quality, and to assess (2) process quality (provider knowledge and ability to apply clinical guidelines; patient satisfaction, and observation of use of provider time).

#### Step 2: audit and feedback meeting

**Fig. 1:**
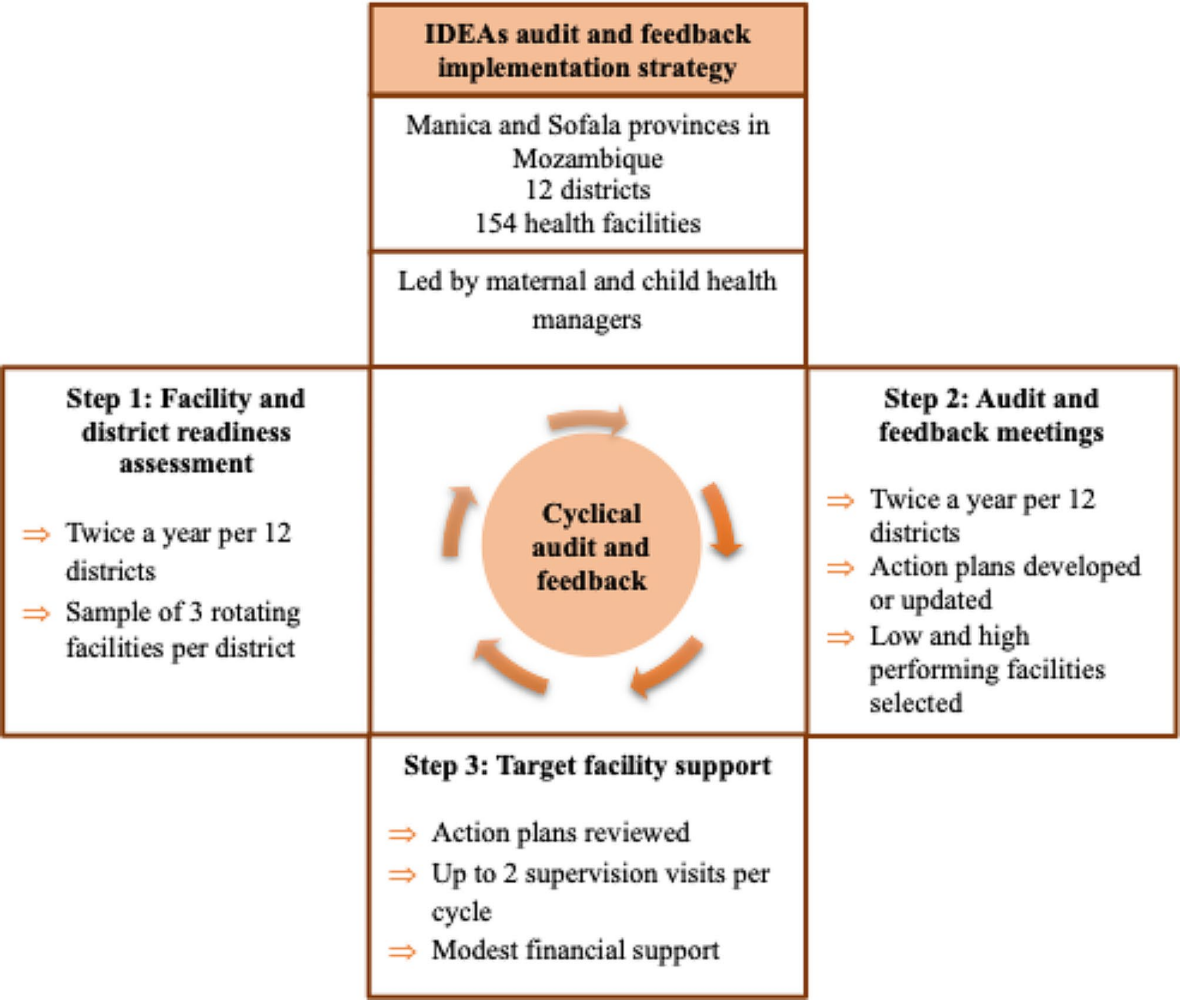
Steps of the IDEAs audit and feedback strategy

MCH managers from the facility, district, and provincial levels participated in semiannual A&F meetings. Auditing data from routine health information systems and SRAs was used to compare performance relative to goals. Feedback was provided in both graphical and tabular formats, allowing visualization of secular trends of service indicators. Each facility and district team presented their performance metrics, followed by a group discussion to interpret results, identify barriers to guideline adherence, and develop action plans highlighting priority problems, specific and measurable targets, and resources required to implement micro-interventions (Fig. 1).

#### Step 3: targeted facility support

At each district A&F meeting, three facilities were selected based on the performance of service delivery indicators (one high-performing and two low-performing) to receive up to two supervision visits per cycle. A modest monthly financial support (US$1,250) was allocated to the district to support action plan implementation in selected health facilities. During supervision visits, action plans were reviewed, barriers to guideline implementation were identified, and technical assistance to address context-specific barriers was provided. Provincial and district MCH managers were responsible for monitoring, evaluating, and recording the degree of success (proportion of micro-intervention implemented successfully) by the health facilities (Fig. 1).

### Study setting

Manica and Sofala provinces are located in central Mozambique and have a combined population of > 4.5 million inhabitants [22, 23]. The IDEAs implementation strategy was implemented in all 154 primary health facilities across 12 districts, representing > 70% of the population in both provinces (Table 1). Intervention districts were selected based on their large population size, robust health facility network, and geographic accessibility to maximize resource investment. Districts were selected as the intervention unit because they are logical disseminating agents, can access resources to meet health facility needs, have the authority to implement management decisions within subordinate health facilities and have a broad reach across the health system.

**Table 1:** IDEAs study setting

Province	Neonatal mortality rate (2019–2020)^a^	IDEAs districts	Population (2021)^b^	District coverage^c^	N° of IDEAs facilities	Health facility coverage^d^
Manica	31 per 1000 live births	Chimoio	456,775	10%	6	2%
		Manica	257,191	5%	17	6%
		Mossurize	230,705	5%	11	4%
		Gondola	224,603	5%	10	4%
		Barue	217,254	5%	13	5%
		Sussundenga	195,258	4%	13	5%
		Vanduzi	130,893	3%	9	3%
Sofala	33 per 1000 live births	Beira	696,515	15%	15	5%
		Nhamatanda	318,380	7%	17	6%
		Dondo	223,484	5%	15	5%
		Buzi	201 710	4%	15	5%
		Gorongosa	202,043	4%	13	5%
		**Total**	**3.354.811**	**71%**	**154**	**56**%

(a) Countrywide surveillance data (provincial estimates). District neonatal mortality rate is not available. The national neonatal mortality rate is 23 per 1000 live births [24];

(b) Provincial Statistical Data 2021; (c) Percentage based on the population of both provinces; (d) Percentage of health facilities based on the total facilities of both provinces. For Manica and Sofala provinces, the combined total population is 4.702.874, and the combined number of health facilities is 277

### Study design

The IDEAs strategy applied a quasi-experimental study design guided by the RE-AIM framework. All dimensions of the RE-AIM framework were used to plan, evaluate, and report the IDEAs strategy. RE-AIM guided the selection and definition of implementation outcomes (reach, adoption, implementation fidelity, and maintenance) reported in the present manuscript and the methodology to evaluate the strategy’s effectiveness on service and readiness outcomes (reported elsewhere). In examining the implementation process, descriptive statistics and qualitative document reviews were used for data extraction and reporting on implementation outcomes. The effectiveness will be assessed using a controlled time series analysis.

### Data analysis: RE-AIM measures

#### Reach

The targeted reach for the IDEAs intervention strategy was 57% of health facilities and 70% of pregnant women and newborns across both provinces. For facilities reached, we divided the number of IDEAs facilities by the total number of public sector primary care health facilities in Manica and Sofala. For the population reached, we use two indicators (first and fourth ANC visits). We divided the total number of ANC visits between 2017 and 2020 in IDEAs facilities by the total number of ANC visits in all facilities in both provinces. These two indicators were selected to describe the first contact of women with the health facility and their retention in care. We used the health information system and project assessments as data sources for selecting facilities and extracting the number of ANC visits.

#### Adoption

We defined adoption as the proportion of districts and facilities that initiated the IDEAs intervention. Our target was that 95% of districts and facilities would adopt the intervention. We used A&F meeting reports and program data to verify the participation of each district and facility.

#### Implementation

We examined the fidelity of the implementation strategy in each district. We used program assessments, action plans, and supervision data to report the following measures: (1) The number and frequency of A&F meeting cycles; (2) The number of participants in A&F meetings; (3) The number and frequency of SRAs; (4) The selection of health facilities based on performance; (5) The number of action plans elaborated; (6) The content of action plans (identified problems and proposed micro-interventions); (7) The number and frequency of supervision visits; (8) Financial support to implement action plans; and (9) The proportion of micro-interventions implemented successfully.

Based on the IDEAs timeline and predetermined intervals between A&F meetings, we expected each district to conduct a series of nine A&F meetings (cycles) between October 2016 and December 2020.

Analyzing action plan content over time was challenging as each facility identified problems relevant to its context, leading to heterogeneity of action plan descriptions. We subsequently grouped similar problems (written differently but with the same meaning or intent), resulting in a list of 41 specific, mutually exclusive problems that micro-interventions were designed to address.

Micro-interventions were initially grouped into six general categories and 23 specific subcategories delineated prior to the study based on a previous pilot experience (Table 2). After removing two unused subcategories from the “health services organization” category, 21 subcategories remain in the final categorization.

**Table 2:** Categories and subcategories of micro-interventions proposed in action plans

General category	Specific subcategory
**Information, Education, and Communication (IEC) for health**	1. Education of patient’s activities
	2. Community education activities
	3. All other activities related to IEC for patients
**Health services organization**	4. Activities related to changes in patient flow
	5. Activities related to changes in service schedules
	6. All other activities contributing to the improvement of health facilities organization
**Commodities stock management**	7. Actions to improve medicine management
	8. Actions to improve management of medical and surgical supplies
	9. All other activities related to the management of materials and goods for the regular operation of health facilities
**Information system management**	10. Data record improvement activities
	11. Data quality audit activities
	12. Health information system file improvement activities
	13. Activities related to the regular sending of information
	14. All other activities contributing to improvements in the availability, quality, and use of data
**Inter- and intra-institutional coordination and collaboration**	15. Intra-institutional coordination activities
	16. Actions to strengthen compliance with clinical standards and protocols
	17. Actions related to the discussion of clinical cases and institutional deaths
	18. On-the-job training activities
	19. All other activities contributing to the quality of healthcare
**Community involvement**	20. Coordination and collaboration activities with the community
	21. Community involvement in the management and organization of healthcare services

#### Maintenance

Three time points were predetermined to evaluate maintenance: 12, 24, and 54 months. We measured the proportion of health facilities sustaining the IDEAs strategy as designed (i.e., holding A&F meetings at least semiannually) at 12 and 24 months and the proportion of health facilities completing meetings at 54 months. Our target was for 90% of health facilities to have implemented the intervention at 12 months, more than 80% at 24 months, and more than 70% completing nine cycles of meetings at 54 months. We counted the months between the first and last A&F meetings for each district and facility and evaluated their participation at each time point.

## Results

### Reach

Facilities: 277 public primary health care facilities were registered in the health information system in both provinces during the study period. Of those, 154 facilities (56%) were exposed to the IDEAs intervention.

Population: 698,683 pregnant women attended the first, and 491,536 attended the fourth ANC visit between January 2017 and December 2020 at IDEAs facilities, compared with 318,253 and 183,933 in non-intervention facilities in Manica and Sofala province, indicating that 69% and 73% of pregnant women and newborns received first and fourth ANC visits in facilities exposed to the IDEAs strategy.

### Adoption

Adoption was 100%, with all 154 health facilities in the 12 districts initiating A&F meetings. However, 13 health facilities in Barue district initiated meetings in the second cycle (out of nine possible cycles), and four facilities in Beira, Gondola, and Vanduzi districts initiated meetings even later (in the seventh and eighth meeting cycles).

### Implementation fidelity measures

#### Audit and feedback meetings

One hundred-seven district A&F meetings occurred during the study period, out of an expected 108. Of the 154 facilities included in this study, 109 (71%) completed all nine A&F cycles, while 45 (29%) completed fewer than nine (34 conducted eight, seven conducted seven, two conducted two, and two conducted one cycle).

Based on the number of facilities and participants from multiple health system levels (facility, district, province), the program expected a minimum of 1512 participants over nine cycles. However, 3076 MCH managers participated in A&F meetings during the study period. Of these, 64% (*n* = 1954) were from health facilities, 29% (*n* = 905) were from district management teams, and 7% (*n* = 217) were from the provincial health departments.

The average interval between meetings was 5.9 months (ranging from 5.3 in Barue to 6.2 in Buzi district), close to the target interval of six months. The duration of each meeting was five days, with the first three days reserved for preparation (data organization, performance matrix elaboration, and presentation rehearsals) and the remaining two days for presentations, discussions of service performance, and elaboration of action plans.

#### Frequency of health service readiness assessments

The goal was to assess readiness in 36 facilities per semiannual cycle in a rotating approach. In practice, there were delays in starting the assessments, and the original sample of 36 was reduced to 24 facilities (2 facilities per district per cycle). By the end of the study, 52% (*n* = 168) of the expected facilities were assessed.

#### Selection of health facilities based on performance

Per protocol, during each A&F meeting, the district selected three health facilities—one high-performing and two low-performing—for a total of 321 selections (214 low-performing, 107 high-performing) across the implementation period. By the end of the study period, 128 of the 154 health facilities (83%) had been selected at least once (some were selected multiple times over the nine cycles), totaling 309 (96% of expected) selections (*n* = 206 low-performing, *n* = 103 high-performing). The number of times each facility was selected during the study varied from zero to seven. Thirty-seven health facilities switched from high-performing to low-performing, or vice versa, between cycles; 28 were selected as high-performing, 63 as low-performing, and 26 were not selected at any point.

#### Elaboration of action plans and identification of problems and micro-interventions

Based on the number of health facilities participating in each A&F meeting, we expected 1294 action plans to be developed. By the end of the study period, 1257 (97% of expected) action plans had been developed.

**Fig. 2:**
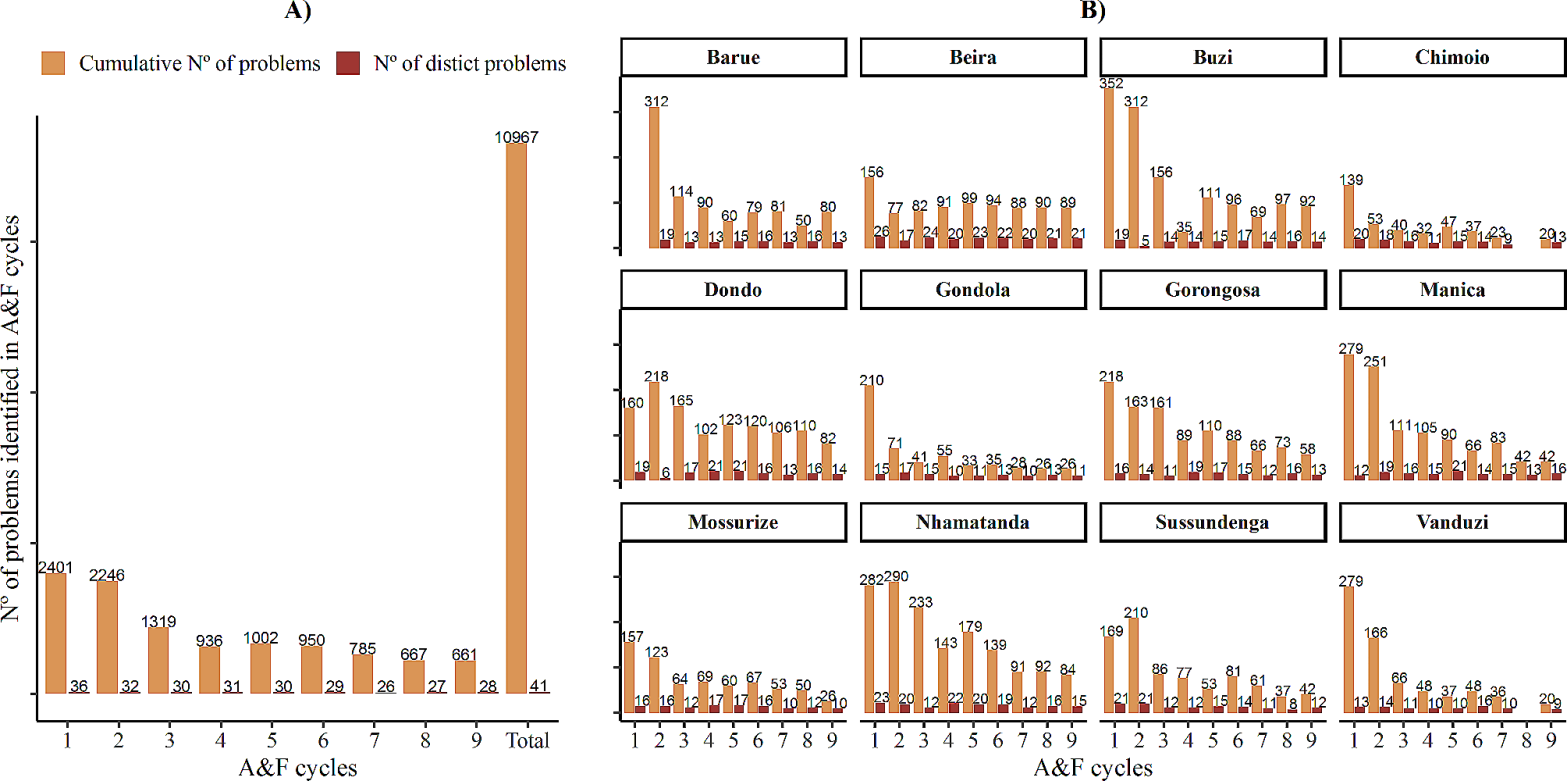
Number of problems identified in audit and feedback. (A) Overall and (B) By district

A total of 10,967 problems were identified in all 12 districts during the nine A&F meeting cycles (Fig. 2A). After grouping these problems based on similarity, we developed a list of 41 distinct problems identified by facility managers. Most problems were identified during the first two cycles, with a drop in the number in subsequent cycles (Fig. 2A and B). Facilities participating in each district A&F meeting tended to identify the same problems, as seen by the difference between the cumulative number and number of distinct problems (Fig. 2).

Nhamatanda, Buzi, and Dondo districts in Sofala province identified the highest proportion of problems (14%, 12%, and 10%, respectively), and Mossurize, Gondola, and Chimoio districts in Manica province identified the lowest proportion (6%, 5%, and 4%, respectively).

In our analysis of the distribution of problems by MCH services, the majority of problems identified were related to antenatal care (46%), followed by maternity (24%), at-risk child consultations (13%), family planning (10%), and postpartum (7%) services.

**Fig. 3:**
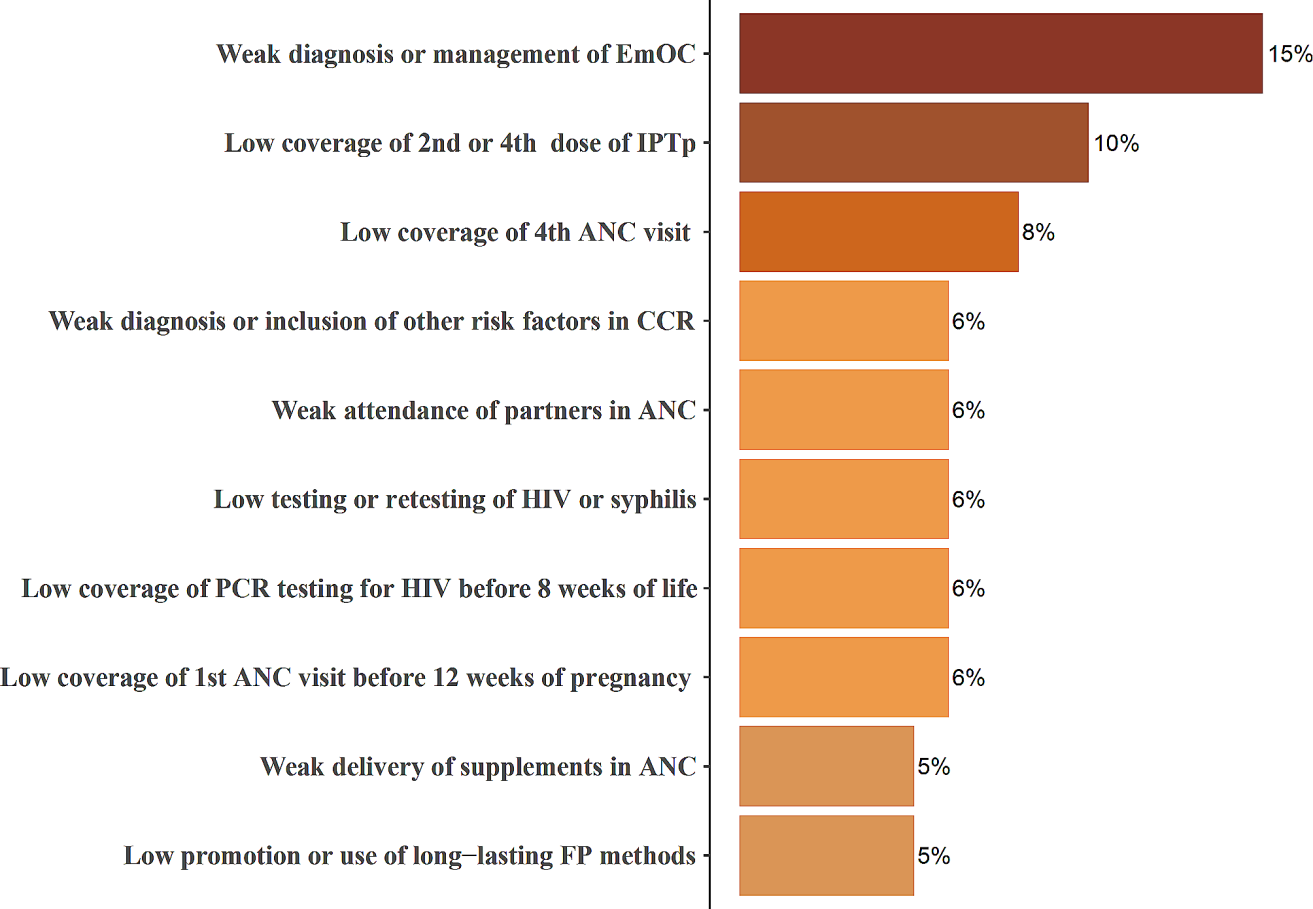
Rank of ten priority problems identified in A&F meetings. Note Fig. 3: EmOC: emergency obstetric complications; IPTp: intermittent preventive treatment for malaria; CCR: *consulta da criança de risco* (“at-risk child consultation” in Portuguese); ANC: antenatal care; PCR: polymerase chain reaction; FP: family planning

When ranking all 41 distinct problems, the cumulative proportion of the top 10 was 73%, as illustrated in Fig. 3.

The following descriptive analysis focuses on the top 10 ranked problems by district and by cycles of A&F.

The top problem consistently identified in eight of the 12 districts and six of the nine cycles was “weak diagnosis or management of EmOC”. When it was not identified as the main problem in a district, it was substituted with “low coverage of the fourth ANC visit,” “low coverage of the first ANC visits before 12 weeks of pregnancy,” or “low coverage of the second or fourth dose of IPTp,” respectively. In cycles where weakness in EmOC was not the main issue, “low coverage of the first ANC visits before 12 weeks of pregnancy” was the substitute.

While most problems were identified in multiple districts and cycles, some were more restricted. For instance, “lack of sphygmomanometer” was listed as a priority only in Chimoio district and during the first cycle. Similarly, “weak diagnosis or management of neonatal asphyxia” was identified as a priority only in Beira and the second cycle.

#### Overall proposed micro-interventions

**Fig. 4:**
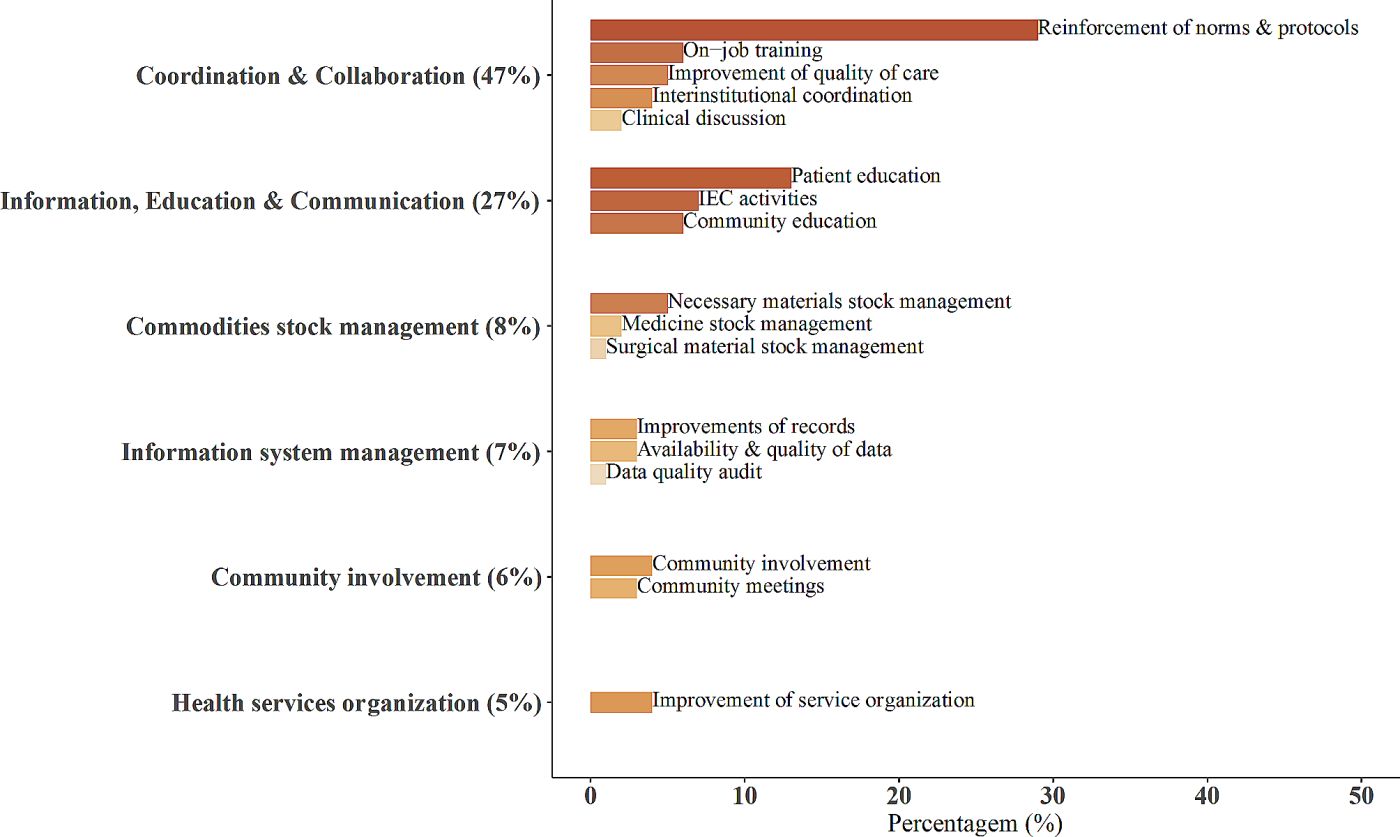
Distribution of general categories and subcategories of micro-interventions proposed in A&F meetings. Note Fig. 4: The following four subcategories with less than 1% were omitted in the figure: “regular sending of data,” “patient flow,” “health information system file improvement,” and “changes in service schedules.”

The micro-interventions proposed in the action plans were categorized into six predefined general categories and ranked as follows: Inter- and intra-institutional coordination and collaboration (47%); Information, Education, and Communication (IEC; 27%); commodity stock management (8%); information system management (7%); community involvement (6%); and organization of health services (5%) (Fig. 4). In addition, 21 subcategories were predefined. Within these subcategories, “reinforcement of norms and protocols (29%)” was the top selection, followed by “patient education (13%)” (Fig. 4).

Table 3 shows the distribution of categories of micro-interventions proposed to address the top 10 problems identified. Micro-interventions in the IEC category were selected primarily to address “weak attendance of partners in ANC”. In contrast, micro-interventions in the intra- and inter-institutional coordination and collaboration category were mainly selected to address “weak diagnosis or inclusion of other risk factors in CCR” and “weak diagnosis or management of EmOC”. Participants selected community involvement activities to address issues such as “low coverage of the first ANC visits before 12 weeks of pregnancy” and “low promotion and use of long-lasting family planning methods”. The health services organization category was primarily selected to address the “weak attendance of partners in ANC” and the “weak diagnosis or management of EmOC”. Improvements in the management of commodities stock were mainly proposed to address “low coverage of the second or fourth dose of IPTp” and “weak delivery of supplements in ANC”.

**Table 3:** Distribution of micro-interventions proposed to address the top 10 problems

General Category & Subcategory of micro-interventions	Top 10 problems identified in A&F meetings									
	1. Weak diagnosis/management of EmOC	2. Low coverage of 2nd/4th dose of IPTp	3. Low coverage of 4th ANC	4. Low testing/ retesting of HIV or syphilis	5. Low coverage of 1st ANC < 12 weeks	6. Weak diagnosis/inclusion of other risk factors in CCR	7. Weak attendance of partners in ANC	8. Low coverage of PCR < 8 weeks of life	9. Low promotion/use of long-lasting methods of FP	10. Weak delivery of supplements in ANC
**Information, Education & communication**	**4%**	**18%**	**47%**	**11%**	**53%**	**7%**	**70%**	**35%**	**67%**	**6%**
a. Education of patient’s activities	48%	50%	52%	5%	26%	92%	18%	98%	55%	63%
b. Community education activities	32%	20%	22%	4%	49%	4%	18%	1%	20%	13%
c. Other IEC activities for patients	20%	30%	26%	91%	25%	4%	64%	0%	25%	23%
**Health Services Organization**	**8%**	**1%**	**2%**	**1%**	**2%**	**1%**	**10%**	**1%**	**2%**	**1%**
a. Activities related to changes in patient flow			6%	11%	13%		72%		10%	
b. Activities related to changes in service schedules		9%								
c. Other activities to improve facility organization	100%	91%	94%	89%	88%	100%	28%	100%	90%	100%
**Commodities stock management**	**2%**	**16%**	**0%**	**7%**	**0%**	**0%**	**1%**	**2%**	**5%**	**14%**
a. Actions to improve medicine management	6%	30%	75%	6%		50%	83%		17%	69%
b. Actions to improve surgical supplies management	14%								79%	
c. Other activities related to the management of materials and goods for the regular operation of the facility	80%	70%	25%	94%	100%	50%	17%	100%	3%	31%
**Information system management**	**4%**	**25%**	**5%**	**2%**	**2%**	**2%**	**2%**	**4%**	**1%**	**29%**
a. Data record improvement activities	79%	40%	36%	31%	31%	57%		59%	67%	42%
b. Data quality audit activities	5%	12%	4%	6%	6%	29%	25%		33%	20%
c. HIS file improvement activities		1%								1%
d. Activities related to the regular sending of information		1%	23%							
e. Other activities to improve the availability, quality, and use of data	16%	46%	36%	63%	63%	14%	75%	41%		37%
**Inter- and intra-institutional coordination and collaboration**	**81%**	**36%**	**31%**	**78%**	**17%**	**84%**	**4%**	**53%**	**12%**	**50%**
a. Intra-institutional coordination activities	2%	4%	3%	1%	17%	24%	31%	16%	9%	5%
b. Actions to strengthen compliance with clinical standards and protocols	40%	68%	58%	96%	58%	60%	38%	53%	42%	89%
c. Actions to discuss clinical cases or institutional deaths	18%	0%	1%	0%		0%				0%
d. On-the-job training activities	19%	26%	35%	1%	16%	1%		8%	32%	5%
e. Other activities to improve the quality of patient care (includes additional supervision)	21%	2%	3%	2%	9%	14%	31%	23%	17%	1%
**Community Involvement**	**1%**	**4%**	**13%**	**0%**	**25%**	**5%**	**13%**	**4%**	**13%**	**0%**
a. Coordination and collaboration activities with the community	47%	62%	47%		45%	11%	53%		9%	
b. Community involvement activities in the management and organization of HF	53%	38%	53%	100%	55%	89%	47%	100%	91%	100%

Note Table 3:

Where is 0%: the value is less than 0.5%. IEC: Information, Education, and Communication; IPTp: Intermittent preventive treatment for malaria; PCR: polymerase chain reaction; CCR: at-risk child consultation; ANC: antenatal care; HIS: Health information system; HF: Health facility; EmOC: Emergency obstetric care;

#### Supervision after A&F meetings

Health facilities selected to receive supervision visits were identified during A&F meeting, with each facility receiving up to two supervision visits per 6-month cycle. One hundred-forty-one (92%) health facilities received 535 supervision visits during the study period. Of all supervision visits, 342 (64%) were double visits (first and follow-up), and 36% were single visits. The number of supervision visits varied between 38 in Buzi and 51 in Manica and Sussundenga districts and between zero and ten per facility.

Four of the 128 health facilities selected based on their performance received no supervision visits (one low-performing and three high-performing). Of the 63 health facilities selected as low-performing only, the average number of visits was 3.7 (range, 0 to 10); of the 37 health facilities selected as either low-performing or high-performing, the average number of visits was 5.2 (range, 2 to 10); and of those selected only as high-performing, the average number of visits was 2.6 (range, 0 to 6).

The average time between an A&F meeting and the first supervision visit was 2.4 (1.6 to 4.1) months, and 3.7(2.9 to 4.6) months between a meeting and the second supervision.

#### Financial support to implement action plans

A monthly amount of $1,250 was provided to each district to support implementing action plan activities in selected facilities. All districts received this support as planned. Districts were responsible for allocating resources to health facilities according to their perceived priority in terms of needs.

#### The proportion of micro-interventions successfully implemented

By the end of the study period, 1851 (17%) micro-interventions were recorded as being implemented successfully. Fewer micro-interventions were reported to be implemented successfully early in the process, but this improved substantially over time (ranging from 0.6% in the first cycle to 46% in the eighth cycle). Among districts, the proportion of successfully implemented micro-interventions varied between 7% in Vanduzi and 33% in Dondo. Overall, districts in Sofala presented better reports compared with those of Manica.

### Maintenance

A&F meetings were conducted over 51 months (October 2016 to December 2020), and supervision visits occurred over 54 months (October 2016 to March 2021).

Of the 154 participating health facilities, 96% (*n* = 148) and 95% (*n* = 146) sustained the intervention at 12 and 24 months, respectively. At 54 months, 71% (*n* = 109) had completed all nine A&F meeting cycles.

**Fig. 5:**
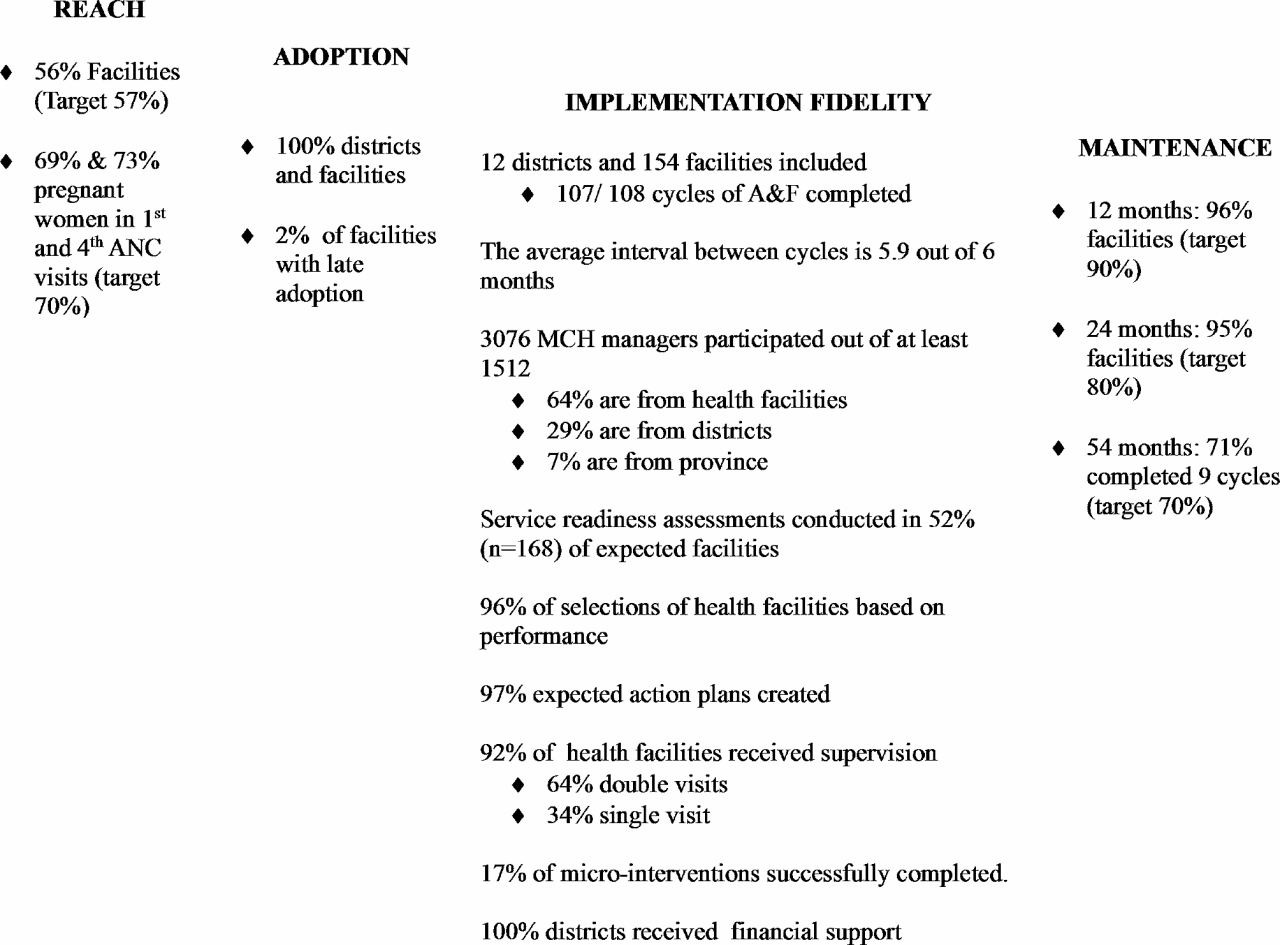
Summary of findings based on four RE-AIM domains

Figure 5 summarizes the findings based on the RE-AIM domains used in this process evaluation.

## Discussion

The IDEAs strategy was designed to support achieving the Sustainable Development Goal target 3.2, focusing on reducing newborn and child mortality. The strategy utilized MCH managers to lead an A&F process aiming to improve the implementation of evidence-based interventions targeting causes of neonatal mortality by identifying and solving facility-level gaps related to the delivery of services according to MOH guidelines. The number of districts and facilities exposed and adopting IDEAs was close to the proposed targets, indicating that the program was successfully introduced and implemented in most primary healthcare facilities across 12 Manica and Sofala provinces districts.

Most facilities completed the intended nine cycles of A&F meetings. However, a handful of facilities did not complete all cycles due to a variety of reasons, such as competing activities during scheduled A&F meetings that prevented MCH managers from participating (e.g., health campaigns, MOH visits, or other supervision visits), as well as unavailability of MCH managers due to vacations, maternity leave, and sickness.

Notably, the participation of MCH managers was high across all levels (provincial, district, and facility) in all 107 cycles, and most participants were from the facility level. This distribution in participation was important because changes needed to improve guideline compliance focus on the service delivery (facility) level. The presence of district and provincial managers was essential for reinforcing the accountability of healthcare providers and harmonizing operational recommendations to implement action plan activities, as described in a separate qualitative study conducted at an earlier stage of the strategy [25].

Positive intervention fidelity was also verified in the duration and frequency of meetings for most districts. However, Barue and Mossurize districts presented the shortest average intervals between meetings (5.3 and 5.5 months, respectively), which could potentially reduce opportunities to implement action plan activities.

The selection of facilities based on performance to receive supervision visits and financial support also had high fidelity, as most facilities had the opportunity to receive supportive supervision.

Identifying and specifying problems and micro-interventions improved over time as part of the action-planning process. A higher number of problems were identified in the first two cycles, and poorly specified micro-interventions were proposed during this period, posing challenges in monitoring their implementation. As a result, around the fourth cycle, the IDEAs team recommended that district teams limit the number of problems per facility to five to improve problem prioritization, specification, and implementation of micro-interventions.

Many identified problems were repeated across different facilities within the same district. This repetition could be attributed to the fact that health facilities faced similar challenges, or it could be due to the group setting of the meetings, which may have influenced participants to think similarly when developing action plans.

The consistent identification of the same problems across multiple meeting cycles suggests that these issues persisted or that the definition of the problem was too broad. For example, the “weak diagnosis or management of EmOC” issue was prioritized in all nine A&F cycles when data from all districts were aggregated. However, different facilities may have observed varying degrees of weaknesses in different components of EmOC. While some issues might have been partially addressed, these levels of detail were not reflected in the listed problems. A better specification of the problems and examination of specific indicators are necessary to better understand progress in addressing these issues. Conversely, identifying a problem in just one cycle may indicate that the issue was resolved or that other problems were deemed a higher priority in subsequent cycles.

We also analyzed whether alignment existed between the identified problems, proposed micro-interventions, and the existing literature or formal guidelines and found successful alignment. For instance, the main problem identified in A&F cycles was weak diagnosis and management of obstetric complications, a well-documented weakness in Mozambique [26, 27] as in many other low-resource settings [28, 29]. Specific actions written in action plans to address this issue included reinforcing the use of partograms to monitor labor, recording the entire case history of patients, conducting complete physical examinations of all pregnant and postpartum women upon entry into the maternity ward and before discharge, and providing on-the-job training in EmOC. These actions are all recommended in national norms for childbirth, newborn care, and obstetric complications in Mozambique [30].

Poor fidelity was observed in conducting semiannual SRAs before A&F meetings and in reporting micro-interventions implemented successfully. Readiness assessment delays resulted from challenges in elaborating the assessment protocol, delays in IRB approval, and failure to rapidly synthesize results to feed back into the A&F meeting after conducting the readiness assessment. Given the delays and high cost, this component was not supported throughout the study. We believe that the impact of not having SRA before each A&F meeting was minimal because MCH managers were knowledgeable about the availability of resources based on other sources, such as supervision visits and routine facility reports.

Reporting the extent to which micro-interventions were completed was sub-optimal, especially at the beginning of the program, due to challenges in monitoring the large number of poorly specified micro-interventions. Additionally, comparing this indicator between the two implementing provinces found better documentation of micro-intervention implementation in Sofala than in Manica province, suggesting that MCH managers from these provinces had different levels of experience in evaluating action plan implementation. One possible explanation for the differences could be that Sofala had prior experience with a similar implementation strategy piloted before the expansion to include Manica province [31].

Quality improvement strategies for maternal and child health have been studied in other developing countries [32, 33]. For A&F in particular, the bulk of evidence available reports on the effectiveness, mostly from high-income settings where the magnitude of the effect varied between a -17–49% improvement in professional practice and null effects for improvements in distal health outcomes [16, 34]. While we agree that it is essential to assess the effectiveness of new strategies, we want to highlight in this manuscript the need for evaluating and reporting implementation processes of A&F in practice in a resource-constrained setting. By gaining this knowledge, we can develop data-driven and contextual strategies to enhance service delivery and quality of care. For instance, in the case of the IDEAs strategy, we identified priority problems, including weaknesses in clinical practice and challenges in implementing micro-interventions, indicating that other well-known bottlenecks affecting health services need to be studied and targeted, including health workers shortages, clinical skills gaps in the management of care, absence or stock-outs of essential commodities and supplies, weak leadership, and inadequate resource allocation [35] and that other strategies, such as training of nurses in EmOC and direct funding of health facilities instead of districts, should be considered to add to the A&F strategy.

This evaluation has several limitations. First, it is essential to note that no statistical inference is made as the evaluation focuses solely on describing the implementation process and outcomes; the strategy’s effectiveness in improving availability and quality of services will be reported in a separate manuscript. Second, population reach was evaluated using indirect indicators of pregnant women using antenatal services. Third, our approach to categorizing problems and micro-interventions based on predetermined categories could potentially obscure important details about these components. Lastly, this study took place in only two provinces of central Mozambique, and therefore, it may not be appropriate to generalize the results to other locations. Despite these limitations, the study also has notable strengths. We applied the RE-AIM framework to plan, evaluate, and report the implementation process and impact of the IDEAs strategy. RE-AIM is a well-recognized and widely used framework in implementation science, and its use provides clarity on how strategies are being designed and evaluated, allowing meaningful comparisons between similar studies.

Additionally, the detailed description of a 4.5-year implementation process and its outcomes enabled highlighting the perspectives of MCH managers related to priority challenges and strategies to overcome them, as well as to detect weaknesses in the components of the IDEAs strategy in responding to those challenges. This information is valuable in recommending refinements or adaptations to improve implementation fidelity and in discussing the strategy’s potential impact on improving health outcomes. Moreover, the cyclical nature of the IDEAs strategy offered an opportunity to build the capacity of MCH managers in Manica and Sofala in using data for learning and decision-making and in proposing tailored evidence-based interventions towards improvements of readiness and quality of care, contributing to the global effort of improving health service delivery to reduce neonatal mortality.

Based on the lessons in implementing IDEAs, we recommend refinements of the strategy components to improve fidelity in implementation, namely, remove the semiannual SRA component or reduce its frequency and only assess a sub-sample of SRA items that are related to indicators examined in the A&F meetings. This approach would improve practicality and reduce the costs of conducting SRAs. In the A&F meetings, each facility should elaborate its action plan separately from other facilities to avoid potential replication in identified problems and solutions, as the definition of problems and micro-interventions need to be specific to the individual health facility context. Additionally, it is crucial to explore the challenges in implementing micro-interventions, specifically the availability of resources and clinical competencies. In the IDEAs case, understanding the impact of funding to the districts to support health facility action plan implementation and reasons for weakness in clinical practice are essential to improve the design of the strategy or identify other strategies that can be added to A&F.

## Conclusion

This report demonstrates that MCH managers can lead Audit and Feedback processes in primary health care in Mozambique. The IDEAs program reach, adoption, and maintenance were aligned with targets. Implementation fidelity was optimal in most components except conducting semiannual readiness assessments and evaluating micro-intervention completion. These components of the intervention should be refined to improve fidelity. Hopefully, our findings and recommendations can inform future replication, adaptation, or scale-up of A&F strategy in Mozambique or similar settings.

### Electronic supplementary material

Below is the link to the electronic supplementary material.

Supplementary Material 1

Supplementary Material 2

Supplementary Material 3

## Data Availability

The data supporting this study’s findings are available upon reasonable request from the corresponding author and with permission of the MOH, Manica, and Sofala provincial health directorate.

## Abbreviations

A&F: Audit and feedback
ANC: Antenatal care
ARV: Antiretroviral
CCR: *Consulta da criança em risco* (“at-risk child consultation” in Portuguese)
EmOC: Emergency obstetric care
FP: Family planning
IDEAs: Integrated District Evidence to Action program
IEC: Information, Education, and communication
IPTp: Intermittent preventive treatment for malaria
MCH: maternal and child health
MOH: Ministry of Health
PCR: Polymerase chain reaction
RE-AIM: Reach, Effectiveness, Adoption, Implementation, and Maintenance
SRA: Service readiness assessments
