# Supplementary material for: Implementation outcomes of the integrated district evidence to action (IDEAs) program to reduce neonatal mortality in central Mozambique: an application of the RE-AIM evaluation framework
Source: BMC Health Serv Res. 2024 Feb 2;24:164. doi: 10.1186/s12913-024-10638-4 (PMC10835896; doi:10.1186/s12913-024-10638-4)
Supplement: Supplementary file 1 — Supplementary Material 1 [file 12913_2024_10638_MOESM1_ESM.docx]

**Additional file 1**: Ranked list of 41 specific problems identified in IDEAS audit and feedback strategy in 12 districts of Manica and Sofala Provinces in Mozambique between October 2016 to December 2021.

| **Rank** | **Distinct problems** | **N** | **%** |
| --- | --- | --- | --- |
| 1 | Weak diagnosis or management of obstetric complications | 1698 | 15.5 |
| 2 | Low coverage of second or fourth dose of IPTp | 1089 | 9.9 |
| 3 | Low coverage of fourth ANC | 867 | 7.9 |
| 4 | Low testing or retesting of HIV or syphilis | 705 | 6.4 |
| 5 | Low coverage of first ANC <12 weeks | 687 | 6.3 |
| 6 | Weak diagnosis or inclusion of other risk factors in CCR (tuberculosis, prematurity, twins, malnourished) | 667 | 6.1 |
| 7 | Weak attendance of partners in ANC | 630 | 5.7 |
| 8 | Low coverage of PCR <8 weeks | 620 | 5.7 |
| 9 | Low promotion or use of long-lasting methods | 528 | 4.8 |
| 10 | Weak delivery of supplements (vitamin A, ferrous salt, mebendazole) | 508 | 4.6 |
| 11 | Poor screening of cervical or breast cancer | 387 | 3.5 |
| 12 | Weak delivery of prophylaxis in newborns (vitamin K, chlorhexidine, eye drops, antiretroviral therapy) | 289 | 2.6 |
| 13 | Low coverage of first or third+ postpartum | 258 | 2.4 |
| 14 | Lack of childbirth and newborn care material (delivery, resuscitation kits/ bed protectors/family planning kits) | 218 | 2.0 |
| 15 | Increase cases of low birth weight/prematurity | 203 | 1.9 |
| 16 | Weak delivery of bed nets in ANC | 201 | 1.8 |
| 17 | Weak diagnosis or management of neonatal asphyxia | 190 | 1.7 |
| 18 | Low coverage of family planning/new users | 170 | 1.6 |
| 19 | Increase of malaria in pregnancy | 145 | 1.3 |
| 20 | Lack of sphygmomanometer | 124 | 1.1 |
| 21 | Weak delivery of prophylaxis in pregnancy (isoniazid, cotrimoxazole, antiretrovirals) | 123 | 1.1 |
| 22 | Low coverage of institutional delivery | 121 | 1.1 |
| 23 | Weak management of syphilis in pregnant women/partner | 121 | 1.1 |
| 24 | Data discrepancy/poor record/ report/interpretation | 90 | 0.8 |
| 25 | Increase in deliveries outside of health facilities | 89 | 0.8 |
| 26 | Low coverage of MCH indicators/vaccines | 70 | 0.6 |
| 27 | Increase cases of malnutrition/HIV in children | 42 | 0.4 |
| 28 | Stockouts (e.g., IPTp, antiretrovirals, penicillin, oxygen, tests, bed nets, chlorhexidine) | 30 | 0.3 |
| 29 | Poor management of transferences | 20 | 0.2 |
| 30 | Low collection of samples for viral load | 15 | 0.1 |
| 31 | Lack of maintenance/adequate infrastructure | 13 | 0.1 |
| 32 | Low coverage of CCR visits | 13 | 0.1 |
| 33 | Poor adherence/follow-up in waiting house | 13 | 0.1 |
| 34 | Lack of classification of action plans | 5 | 0.0 |
| 35 | Poor intersectoral communication | 5 | 0.0 |
| 36 | Weak screening of health conditions in schools | 3 | 0.0 |
| 37 | Increase in sexually transmitted infections in pregnancy | 2 | 0.0 |
| 38 | Increase teenage pregnancy | 2 | 0.0 |
| 39 | Lack of in-service training | 2 | 0.0 |
| 40 | Lack of quality assessments on HIV, syphilis, and malaria tests | 2 | 0.0 |
| 41 | Low number of exclusively breastfed children | 2 | 0.0 |
|  | Total | 10,967 | 100.0 |
| Note: IPTp: intermittent preventive treatment for malaria, ANC: antenatal care, PCR: polymerase chain reaction; ARV: antiretroviral; MCH: maternal and child health; CCR: at-risk child consultation | | | |
